# Supplementary material for: Traditional medicine consumption in postpartum for HBV-infected women enrolled in the ANRS 12345 TA PROHM study in Cambodia
Source: PLoS One. 2023 Aug 10;18(8):e0288389. doi: 10.1371/journal.pone.0288389 (PMC10414559; doi:10.1371/journal.pone.0288389)
Supplement: S1 Table — (PDF) [file pone.0288389.s001.pdf]

Supplementary File: List of plants identified in the study

No	Scientific name	Local name
1	*Saccharum officinarum* L. [Poaceae]	Am pov khmao
2	*Millingtonia hortensis* L. f. [Bignoniaceae]	Ang kea bos
3	*Ziziphus cambodiana* Pierre [Rhamnaceae]	Ang krorng
4	-	Bay nhaenh
5	*Justicia gendarussa* Burm.f. [Acanthaceae]	Chha eung morn
6	-	Chheur krom
7	*Diospyros nitida* Merr [Ebenaceae]	Chheurphlerng
8	-	Chi
9	*Ocimum basilicum* L. [Lamiaceae]	Chi Neang Vorng
10	-	Chorn tul pong morn
11	*Casearia grewiaefolia* Vent. [Flacourtiaceae]	Chroy/Chrouy
12	*Bauhinia pulla* Craib [Fabaceae]	Chun Der Sva
13	*Allophyllus serrulatus* Radlk. [Sapindaceae]	Dai rohat
14	*Piper retrofractum* Vahl [Piperaceae]	Dei phlei
15	*Ceiba pentandra* (L.) Gaertn. [Malvaceae])	Derm Kor
16	-	Derm Pang
17	*Cocos nucifera* L. [Arecaceae]	Dong
18	*Cassia tora* L. [Fabaceae]	Donghert chhneang/ Donghert khmoch
19	*Antidesma ghaesembilla* Gaertn. [Phyllanthaceae]	Dongkeab kdam
20	*Cochlochspermum religiosum* (L.) Alston [Cochlospermaceae]	Kabas prei
21	*Careya arborea* Roxb. [Lecythidaceae]	Kan dorl
22	-	Kderl mnors
23	*Caesalpinia sappan* L. [Fabaceae]	Khlerm sbaeng
24	*Mitragyna speciosa* Korth. [Rubiaceae]	Khtom
25	*Mitragyna parvifolia* (Roxb.) Korth [Rubiaceae]	Khtom phnom
26	*Phanera bassacensis* (Pierre ex Gagnep.) de Wit [Fabaceae]	Klaeng por
27	*Zingiber officinale* Roscoe [Zingiberaceae]	Knhei phlerng/ Knhei kream
28	*Stephania rotunda* Lour. [Menispermaceae]	Ko Ma Pich
29	*Euonymus cochinchinensis* Pierre [Celastraceae]	Ko mouy
30	-	Kondab chong eh
31	*Tetracera indica* (Christm. & Panz.) Merr [Dilleniaceae]	Smao Kontuy domrei
32	*Wurfbainia vera* (Blackw.) Skornick. & A.D.Poulsen [Zingiberaceae]	Krervanh
33	-	Krobei trors
34	*Citrus hystrix* DC. [Rutaceae]	Kroch Serch
35	*Scolopia spinosa* (Roxb.) Warb. [Salicaceae]	Krokhob prei
36	-	Krovanh chheur
37	*Vitex negundo* L. [Lamiaceae]	Kun ti
38	*Flueggea virosa* (Roxb. ex Willd.) Royle [Phyllanthaceae]	Leach phtos
39	-	Leay laek/ Neang nuon
40	*Smilax glabra* Roxb. [Smilacaceae]	Merm thnam chen

41	-	Ming moang
42	*Piper nigrum* L. [Piperaceae]	Mrech
43	*Baeckea frutescens* L. [Myrtaceae]	Mrech ton saay
44	-	Mtaes Krohorm/ Kao ki
45	-	Pa-nhea ya/ Mok
46	-	Pek chi / Kort sor
47	*Dillenia hookeri* Pierre [Dilleniaceae]	Phlou bat
48	*Dillenia ovata* Wall. ex Hook.f. & Thomson [Dilleniaceae]	Phlou thom
49	*Hymenocardia punctata* Wall. ex Lindl. [Phyllanthaceae]	Phnom phnaenh
50	-	Pkar krohorm/ Ang houy
51	*Illicium verum* Hook.f. [Schisandraceae]	Poch kak
52	*Croton caudatus* Geiseler [Euphorbiaceae]	Pro bouy
53	-	Pro phenh
54	*Amphineurion marginatum* (Roxb.) D.J.Middleton [Apocynaceae]	Prolao porpae
55	*Putranjiva roxburghii* Wall. [Putranjivaceae]	Prom
56	*Zingiber purpureum* Roscoe [Zingiberaceae]	Pun Ley
57	-	Reussey prei
58	-	Roleay toch
59	*Pandanus tectorius* Parkinson ex Du Roi [Pandanaceae]	Romchaek
60	*Lasianthus fordii* Hance var. *fordii* [Rubiaceae]	Ror Leay Chheam
61	-	Ruk Reu
62	*Lygodium circinnatum* (Burm.f.) Sw. [Schizaeaceae]	Rum Say Sok
63	*Premna cambodiana* Dop. var *membranacea* P.Dop [Verbenaceae]	Sang kae phlerng
64	-	Sangkhor
65	*Passiflora foetida* L. [Passifloraceae]	Sav mao prei
66	*Strychnos nux-vomica* L. [Loganiaceae]	Slaeng
67	*Cymbopogon nardus* (L.) Rendle [Poaceae]	Sleuk Krey
68	*Cynodon dactylon* (L.) Pers. [Poaceae]	Smao chenh chean
69	*Aphyllodium biarticulatum* (L.) Gagnep. [Fabaceae]	Srae mour
70	-	Srov kro ob/ Seav huy
71	-	Ta kor/ Kro kpr
72	*Cinnamomum cambodianum* Lecomte [Lauraceae]	Tep pirou
73	*Holarrhena pubescens* Wall. ex G.Don [Apocynaceae]	Teuk dos khlar thom
74	*Holarrhena curtisii* King & Gamble [Apocynaceae]	Teuk dos khlar toch
75	-	Teuk Dos Kla Thom
76	*Camellia sinensis* (L.) Kuntze [Theaceae]	Theh
77	*Bridelia ovata* Decne. [Phyllanthaceae]	Thmenh trei
78	*Croton joufra* Roxb. [Euphorbiaceae]	Tom pong
79	*Uvaria dulcis* Dunal [Annonaceae]	Treal sva
80	*Carallia brachiata* (Lour.) Merr. [Rhizophoraceae]	Tro maeng
81	*Diospyros oblonga* Wall. ex G.Don [Ebenaceae]	Tro yeurng
82	*Salacia chinensis* L. [Celastraceae]	Veay
83	-	Vorl Chheam
84	*Celastrus paniculatus* Willd. [Celastraceae]	Vorl Chort

85	-	Vorl Dok peay
86	*Tetracera indica* (Christm. & Panz) Merr. [Dilleniaceae]	Vorl dos kun
87	*Albizia myriophylla* Benth. [Fabaceae]	Vorl Em/Kam Chao
88	-	Vorl kro ob
89	-	Vorl Krobey chul dei
90	*Illigera rhodantha* Hance [Hernandiaceae]	Vorl kroch
91	*Willughbeia edulis* Roxb. [Apocynaceae]	Vorl kuy
92	*Arcangelisia flava* (L.) Merr. [Menispermaceae]	Vorl Romeat
93	*Bridelia retusa* (L.) A. Juss. [Phyllanthaceae]	Vorl Tmenh Trey
94	-	Vorl totong
95	-	Vorl tro ann
96	-	Khtom thom
97	*Nelumbo nucifera* Gaertn. [Nelumbonaceae]	Chhouk
98	*Sesamum indicum* L. [Pedaliaceae]	La-ngor
99	*Curcuma longa* L. [Zingiberaceae]	Romeart
100	*Amaranthus blitum* L. subsp. *Blitum* [Amaranthaceae]	Ptee Ach Moan
101	-	Red clover
102	-	Paraway plus
103	*Achyranthes aspera* L. [Amaranthaceae]	Smav andaat ko
104	*Urceola polymorpha* (Pierre ex Spire) D.J.Middleton & Livsh. [Apocynaceae]/*Aganonerion polymorphum* Pierre ex Spire [Apocynaceae]	Kort prom/Tneng (Vorl)
105	*Allium sativum* L. [Amaryllidaceae]	Ktem sor
106	*Annona squamosa* L. [Annonaceae]	Teab baraing
107	*Bombax ceiba* L. [Malvaceae]	Roka
108	*Bridelia tomentosa* Blume. [Phyllanthaceae]	Tmenh trei
109	*Cordia cochinchinensis* Gagnep. [Boraginaceae]	Bay chreng
110	*Couroupita guianensis* Aubl. [Lecythidaceae]	Raing phnom pka
111	*Cymbopogon nardus* (L.) Rendle [Poaceae]	Slek krei srok
112	*Cyperus rotundus* L. [Cyperaceae]	Smav krovanh chrouk
113	*Dalbergia hancei* Benth. [Fabaceae]	Vorl tos
114	*Dendrocalamus membranaceus* Munro [Poaceae]	Reussei srok
115	*Lagerstroemia floribunda* Jack. [Lythraceae]	Tror bek prei
116	*Leea rubra* Blume [Vitaceae]	Kdaing bay
117	*Loeseneriella dinhensis* (Pierre) A.C.Sm [Celastraceae]	-
118	*Ocimum tenuiflorum* L. [Lamiaceae]	Mreah prov
119	*Phyllanthus cochinchinensis* Spreng. [Phyllanthaceae]	Pro phenh chhmol/Pro phenh slek thom
120	*Phyllanthus taxodiifolius* Beille [Phyllanthaceae]	-
121	*Platycladus orientalis* (L) Franco [Cupressaceae]	Kan tuy domrei
122	*Pouzolzia zeylanica* (L.) Benn. [Urticaceae]	Mouk chhneang
123	*Tetracera sarmentosa* (L.) Vahl. [Dilleniaceae]	-
