# Supplementary material for: Traditional medicine consumption in postpartum for HBV-infected women enrolled in the ANRS 12345 TA PROHM study in Cambodia
Source: PLoS One. 2023 Aug 10;18(8):e0288389. doi: 10.1371/journal.pone.0288389 (PMC10414559; doi:10.1371/journal.pone.0288389)
Supplement: S2 File — (PDF) [file pone.0288389.s003.pdf]

SYNOPSIS OF THE STUDY

ClinicalTrials Id: NCT02937779

Title of Study: Interventional strategy using rapid test screening and TDF treatment for HBV infected pregnant women to prevent HBV mother to child transmission in Cambodia
(ANRS 12345 TA PROHM: Tenofovir As PRevention Of Hepatitis b Mother-to-child transmission)

Short title – Sponsor N°: TA PROHM-ANRS 12345

Sponsor French National Institute for Health and Medical Research-French National agency for Research on AIDS and viral hepatitis **(Inserm-ANRS)**

Coordinating Investigator(s): Dr CHHUN Samsorphea and Dr Olivier SEGERAL

Participating countries: CAMBODIA AND FRANCE

Objectives

Principal objective: Assess the effectiveness of a strategy to prevent HBV mother-to-child transmission (MTCT) in Cambodia based on 1/ the use of rapid tests HBsAg and HBeAg to screen HBV infection 2/ a treatment by TDF, for women with a positive HBeAg test or a negative HBeAg test with ALT > 40 U/L, from 24 weeks of amenorrhea including a "test and treat" strategy for those seen later 3/ early vaccination for all infants at birth (< 2 hours)

Secondary objectives

Describe the HBV viral load (VL) decrease caused by the TDF

Estimate the rate of HBV transmission to newborns according to the time spent on TDF as well as initial viral load level and delivery viral load level

Estimate the rate of HBV transmission to newborns for HBe Ag negative women with ALT < 40 U/L in absence of TDF prevention

Describe the correlation between HBV viral load level and HBe Ag status

Describe subgroups of mothers and new-borns for which the strategy seems more effective

Assess TDF safety in mothers and infants

Analyse the cost-effectiveness of the strategy compare to international guidelines (WHO, APASL)

Methodology: Phase IV multicenter observational and interventional non randomized prospective study.

Expected enrolment 1000 subjects with 295 HBe Ag positive women and 705 HBe negative

Outcomes **Primary outcome:** Proportion of active HBV infection in new-born at 6 months of life estimated by HBs Ag positivity

Secondary outcomes :

- Process and acceptability of the therapeutic intervention:
    - Proportion of women lost to follow up before the last visit of the study
    - Proportion of eligible women accepting the TDF strategy
    - Proportion of women treated by TDF until 6 weeks post-partum among those to whom TDF was initiated
    - Adherence assessment using questionnaires, visual analog scale and pills count
- Proportion of women with viral load > 5.3 Log IU/mL among those HBeAg negative
- Decrease curve of HBV VL after TDF initiation overt time and proportion of women with HBV DNA < 5·3 log10 IU/mL at delivery

- Proportion of new-born with positive HBs Ag at 6 months according to treatment duration, initial and delivery viral load level
- TDF safety among mothers and infants
    - Proportion of women that must stop treatment due to side effects,
    - Proportion of women and/or infants with grade 3 or 4 side effects,
    - Proportion of participant presenting a "flare" event defined by ALT > 5N
- Proportion of women needing treatment continuation in post-partum among those to whom treatment was initiated

---

Eligibility **Inclusion criteria**

- >= 18 years old the day of inclusion
- Pregnancy
- Positive HBs Ag
- Informed consent obtained with information sheet given and explained and the consent form signed by the participant of the project investigator at the latest the day of the inclusion

Non-inclusion criteria

- Women refusing HBs Ag test
- HIV co-infection
- HCV co-infection
- HBV treatment ongoing at the day of inclusion
- Creatinine clearance < 30 mL/min
- Severe gravidic disease present at inclusion involving life threatening to the mother and/or the child including
    - Symptomatic cirrhosis
    - Obstetrical hypertension
    - Rupture of membranes or hospitalization for premature delivery threats
    - Pre-existing maternal cardiopathy
    - All medical conditions life-threatening
- Evidence of pre-existing fetal anomalies incompatible with the child’s life
- Imminent child’s birth defined as cervix dilatation up to 7 centimeters
- Intention to deliver in a maternity not linked to the study
- Any concomitant medical condition that, according to the clinical site investigator would contraindicate participation in the study.
- Concurrent participation in any other clinical trial without written agreement of the two study teams

---

Intervention

Each woman will be informed of the objectives and the total duration of the study as well as the benefits and risks to participate. An information sheet in Khmer will be given to each woman. The study will be composed of two phases of information’s and consent. The first one will be done during the HBs Ag screening using rapid test; the screening will be proposed to all pregnant women attending ANC in one of the affiliated centres. The second phase will be done during the inclusion visit and will concern only HBs Ag positive women. The study will concern only HBs Ag positive women with 1) follow up of all HBsAg positive women from inclusion up to 6 months postpartum 2) For HBeAg positive women or HBeAg negative women with ALT > 40 U/L, initiation of treatment by fumarate de tenofovir disoproxil, with a daily administration of one 300mg pill. Women will be treated from 24 weeks of amenorrhea until 8 weeks post-partum. For women with first ANC after 24 weeks of amenorrhea, treatment will begin the day of inclusion. Treatment will be given for 4 weeks and adherence will be estimated. Supply of TDF will be provided by Ministry of Health and UNICEF and funded by the study.

In all cases, vaccination of the newborn will be carried out according to the national protocol in Cambodia i.e. 4 injections at birth, 6, 10 and 14 weeks of age with the first dose of vaccine provided during the first 2 hours of life.

Statistical methods

The study aims to document the acceptability, the operational implementation of the study. To ensure an observed percentage in a reliable confidence interval of 2% around the value expected of 3% (thus detection of a gap with

regard to 95%CI: 1-5%), a total of 280 women with positive HBe Ag must be evaluated. Considering 5% of women lost to follow-up, a total of 295 HBeAg positive women must be enrolled.

Primary outcome

The percentage, and its 95% confidence interval, of infants with positive HBsAg at 6 months of life will be performed on the available data and stratified on 1/ TDF-eligibility for women 2/ infant's HBIg status, 3/ the duration of TDF for TDF-treated women 4/the amendment of the protocol. Infants lost to follow-up or who withdrew before 6 months will not be included in the primary outcome analysis.
