# Supplementary material for: Traditional medicine consumption in postpartum for HBV-infected women enrolled in the ANRS 12345 TA PROHM study in Cambodia
Source: PLoS One. 2023 Aug 10;18(8):e0288389. doi: 10.1371/journal.pone.0288389 (PMC10414559; doi:10.1371/journal.pone.0288389)
Supplement: Attachment — Submitted filename: Response to Reviewers.docx [file pone.0288389.s004.docx]

*We would like to thank all Reviewers for their careful and thorough reading of this manuscript and for the thoughtful comments and constructive suggestions, which help to improve the quality of this manuscript. Our response to the Reviewers’ comments are in italics.*

**Responses to Reviewer #1:**

**Reviewer #1: This is good work in an area not commonly studied by many researchers and yet herbal Medicine is used quite a lot by women during pregnancy. I have checked and noted that the work has not been published elsewhere before and is written in very good English. The authors have however, not compared the findings to studies of similar nature done in other countries or within their own country if any to put this current one to perspective. it would be a good one to add if the authors don't mind.**

***Reply:***

*We highly appreciate the feedback from the reviewer. We have thoroughly re-performed the literature review. Unfortunately, we haven’t found any studies which assess the toxicity of herbal medicine used during postpartum. However, we have found some articles from other countries mentioning the use of herbal medicine during this particular period. This supports our finding that the postpartum use of herbal medicine is still as common in other places as in Cambodia. We have included the result of the literature review in the Discussion section, page 15, lines 287 and 288 of the revised manuscript.*

**Responses to Reviewer #2:**

**Reviewer #2: In the present study the authors aim to describe traditional medicine consumption during postpartum phase for women enrolled in the TA PROHM study and to investigate the possible association between traditional medicine consumption and acute liver toxicity. As pointed by the authors the study has several limitations, additionally the authors conclude that consumption of remedy with alcohol is associated with hepatocellular injury. However it may not be concluded that the hepatocellular injury is associated with remedy, it may be purely associated with the alcohol content rather then the medicinal plant.**

***Reply:***

*We completely agree with the reviewer. We have by no means associated hepatocellular injury solely to the plants and indeed, hepatocellular injury could be secondary to the consumption of the alcohol alone. However, as alcohol is an inseparable component of the remedy for some specific plants, it is impossible to conclude. In vitro analysis of cytolysis on hepatocytes would be useful to confirm the possible toxicity of these plants. In our article, the terminology “alcohol-based remedies” as a whole was used and associated with the hepatocellular injury.*

*The discussion was completed lines 318 – 321 for that purpose. “Alcoholic maceration of plants or porcupine stomach, as reported in our study, may represent an isolated or a cumulative and exacerbating factor of acute hepatocellular injury for patients with pre-existing risk factor such as HBV infection and exposed to immune restoration. Further investigations are needed to understand whether alcohol alone or the preparation is responsible for this toxicity.”*
